# Experimental Study and Comparison of Imbalance Ensemble Classifiers with Dynamic Selection Strategy

**DOI:** 10.3390/e23070822

**Published:** 2021-06-28

**Authors:** Dongxue Zhao, Xin Wang, Yashuang Mu, Lidong Wang

**Affiliations:** 1 School of Science, Dalian Maritime University, Dalian 116026, China; zhao_yee1128@163.com; 2 School of Artificial Intelligence and Big Data, Henan University of Technology, Zhengzhou 450001, China; muyashuang324@126.com

**Keywords:** dynamic selection, ensemble classification, imbalanced data classification, multi-class classification

## Abstract

Imbalance ensemble classification is one of the most essential and practical strategies for improving decision performance in data analysis. There is a growing body of literature about ensemble techniques for imbalance learning in recent years, the various extensions of imbalanced classification methods were established from different points of view. The present study is initiated in an attempt to review the state-of-the-art ensemble classification algorithms for dealing with imbalanced datasets, offering a comprehensive analysis for incorporating the dynamic selection of base classifiers in classification. By conducting 14 existing ensemble algorithms incorporating a dynamic selection on 56 datasets, the experimental results reveal that the classical algorithm with a dynamic selection strategy deliver a practical way to improve the classification performance for both a binary class and multi-class imbalanced datasets. In addition, by combining patch learning with a dynamic selection ensemble classification, a patch-ensemble classification method is designed, which utilizes the misclassified samples to train patch classifiers for increasing the diversity of base classifiers. The experiments’ results indicate that the designed method has a certain potential for the performance of multi-class imbalanced classification.

## 1. Introduction

Data imbalance is ubiquitous and encountered in the field of classification problems. It occurs when the number of instances for different classes are significantly out of proportion. The minority classes with fewer instances usually contain the essential information, which has been observed in broad application areas, such as medical diagnosis [1,2,3,4,5,6], sentiment or image classification [7,8], fault identification [9,10], etc. Many typical classifiers may generate unsatisfactory results due to a concentration on global accuracy while ignoring the identification performance for minority samples.

It is a significant task to identify minority samples among majority samples to accurately attain essential information. With the rising prominence of machine learning and artificial intelligence, as well as the continuous emergence of new problems and technologies involving imbalanced data, imbalanced classification methods are widely concerned and developed because of its development prospects [11,12,13,14,15,16,17,18]. Improving classification algorithms, introducing cost sensitive strategies, and using balance algorithms are common methods in imbalanced learning.

One of the most widely-used strategies is ensemble learning [19,20,21], which combines classification algorithms with data processing techniques or cost-sensitive solutions. The superiority of ensemble learning in dealing with imbalanced data is that it implements a combined strategy for the classification results based on multiple base classifiers, so that the classifier group can identify a skewed distribution between data categories as much as possible. It has seen great success in improving the identification of minority class samples for imbalanced classification problems. During the training process, the construction way of ensemble classifiers is diverse and flexible. A single classification algorithm can be used to construct homogeneous ensemble classifiers, whereas different classification algorithms can be used to obtain heterogeneous classification systems [22].

The existing body of research on the application of the ensemble model suggests ensemble classification algorithms for imbalanced data have great potential effectiveness in practice [23,24,25,26]. For further improving the classification efficiency, multiple effective ways [27,28] have been developed from several perspectives. In more recent studies, one of the most promising strategies is dynamic selection [29], in which the most competent or an ensemble classifier is selected by estimating each classifier’s competence level in the classification pool. The benefit of this approach is to identify different unknown samples by choosing different optimum classifiers. Therefore, each base classifier can be regarded as an expert for a special sample in the classification space. Dynamic selection classifiers exhibit a higher accuracy over traditional combined approaches in solving several real-world problems, such as face recognition [30] and text verification [31].

Due to a dynamic selection strategy emerging as an interesting technique for extending ensemble algorithms, we are motivated to provide a comprehensive review of the development of the latest ensemble classification algorithms for imbalanced datasets, and offer a detailed experimental comparison of the performance of the state-of-the-art existing ensemble algorithms incorporating dynamic selection. This study is arranged as follows. We collect several sources of imbalanced datasets and discuss several pertinent evaluation indexes in Section 2. In Section 3, we categorically summarize the latest progress in imbalance ensemble algorithms. In an attempt to improve the classification performance for both binary class and multi-class imbalanced datasets, an experimental scheme is designed by adopting a dynamic selection strategy in Section 4. A series of experimental comparisons are conducted to support the role of imbalance ensemble classification with the dynamic selection strategy in Section 5. Section 6 draws conclusions.

## 2. Imbalanced Datasets and Evaluation Measures

In this section, the public access of data sources and comparison criteria used in the related literature are summarized.

### 2.1. Sources of Imbalanced Datasets

Although imbalanced datasets exist widely, most researchers generally select experimental datasets from public access databases to compare the performance of classification models. Table 1 provides a summary of several databases used in the literature.

The UCI database covers 376 datasets used for machine learning classification tasks, including binary class and multi-class datasets. OpenML is an online machine learning platform for sharing or organizing data, machine learning algorithms, and experiments, with a total of 3121 datasets. KEEL includes a module named imbalanced data for imbalance learning containing multi-class imbalanced datasets. In the Defect Prediction database, a series of typical imbalanced datasets concerning network fault detection are available.

### 2.2. Evaluation of Imbalanced Classification

Owing to the skewed distribution of imbalanced data, overall accuracy and precision cannot be enough to measure the recognition performance of the model for the minority classes, especially when the imbalance is extremely high. The G-mean [32] and F-measure [33] are typical evaluations of imbalance learning, which can describe categorization situations from different perspectives. During the experimental process, a comprehensive evaluation relying on multiple indicators should be recruited to examine the effectiveness of a classification model. Suppose *n* is the number of classes, the following performance measures of imbalanced classification are suitable for both binary class and multi-class classification.

(I) MAvA:
  (1) $$\mathrm{MAvA}=\sum_{i=1}^{n}Acc_{i}/n,$$
  where $Acc_{i}$ is the accuracy of the *i*-th class;
(II) G-mean:
  (2) $$G-\mathrm{mean}=\sqrt[{n}]{\prod_{i=1}^{n}Acc_{i}};$$
(III) Precision:
  (3) $$\mathrm{Precision}=\frac{1}{n}\cdot \sum_{i=1}^{n}\frac{{\mathrm{TP}}_{i}}{{\mathrm{TP}}_{i}+{\mathrm{FP}}_{i}},$$
  where ${\mathrm{TP}}_{i}$ denotes the number of correctly classified samples in the *i*-th class, and ${\mathrm{FP}}_{i}$ is the number of instances misclassified into the *i*-th class;
(IV) F-measure:
  (4) $$F-\mathrm{measure}=\frac{2}{n}\cdot \sum_{i=1}^{n}\frac{{\mathrm{Precision}}_{i}\cdot {\mathrm{Recall}}_{i}}{{\mathrm{Precision}}_{i}+{\mathrm{Recall}}_{i}},$$
  where ${\mathrm{Recall}}_{i}=\frac{{\mathrm{TP}}_{i}}{{\mathrm{TP}}_{i}+{\mathrm{FN}}_{i}}$, ${\mathrm{FN}}_{i}$ denotes the number of samples in the *i*-th class which are misclassified into the other class label.

## 3. Ensemble Approaches for Imbalanced Classification

**Figure 1:**
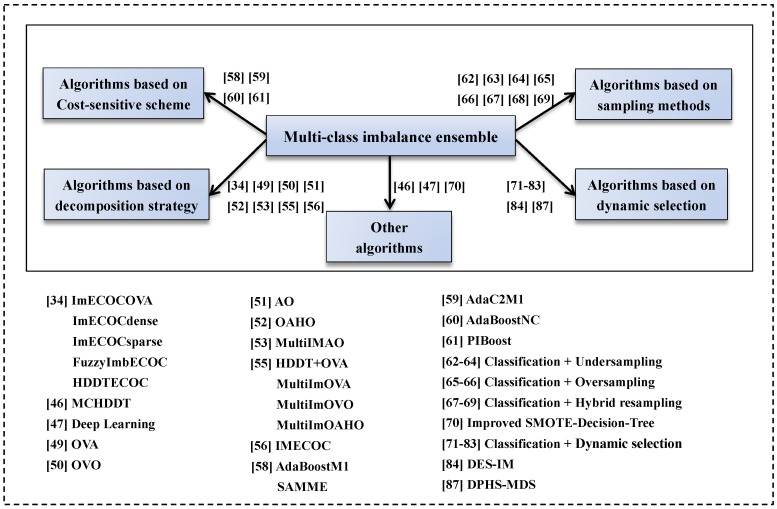
Multi-class imbalance ensemble classification.

The challenges of imbalanced classification and its prevalence have resulted in much research in this area. An effective solution is to design classifiers based on ensemble approaches [34,35,36,37,38,39,40,41,42]. Ensemble schemes for imbalanced classification have been developed from the perspectives of data decomposition, cost-sensitive schemes, sampling methods, and dynamic selection. These methods can achieve desirable results for binary imbalanced classification problems. However, multi-class classification involving multiple minority classes has to face the complexity of the internal structure of multi-class imbalanced datasets, and the differences of a decision boundary of two classes bring more difficulties to the classification task and require additional effort. Therefore, multi-class imbalanced classification [43,44,45,46,47,48] has always attracted attention in the machine learning field. This part summarizes the state-of-the-art imbalance ensemble classification algorithms for binary class and multi-class imbalanced datasets. Some of the pertinent methods are shown in Figure 1 and later elucidated in our comparative study.

### 3.1. Imbalanced Learning Based on Ensemble Classification and a Decomposition Strategy

The most notable characteristic of multi-class classification is the diversity of categories compared with binary classification. A common method is to convert a multi-class dataset into several binary datasets via decomposition, for example, OVA [49], OVO [50], AO [51], and OAHO [52].

OVA is a relatively straightforward decomposition strategy and is designed for disassembling data by marking each category as a positive class and all the other classes as a negative part, on which a classifier is trained. If the original dataset has *n* classes, *n* binary classifiers can be obtained. OVO trains one binary classifier for each pair of classes, a total of n(n−1)/2 classifiers are required to be trained. Based on OVA and OVO, Garcia-Pedrajas et al. [51] proposed the AO strategy, which employs OVA to obtain two predicted results with the label ($l_{i}$, $l_{j}$), and other predicted results from OVO classifiers related to $l_{i}$ and $l_{j}$ are chosen to make the final prediction. OAHO sorts classes according to the number of instances in descending order {$l_{1}$, $l_{2}$, ⋯, $l_{n}$}, where $l_{1}$ is the class which has the largest number of samples. The training process starts from $l_{1}$ until $l_{n}$ and sequentially regards the current class as the positive class and all the lower ranking classes as negative classes. Binary classifiers will be trained on sub-datasets.

Data decomposition is an easily applicable conversion method for multi-class problems and has been combined with ensemble classification in succession. For instance, to address the multi-class imbalance ensemble classification issue, Ghanem et al. [53] combined AO and the PRMS-IM algorithm [54] to design the MultiIMAO classifier. It effectively demonstrated that the data decomposition strategy could enhance classification performance to a certain extent. Besides, Bi et al. [55] incorporated the OVA, OVO, and OAHO methods in PRMs-IM classification, named MultiIMOVA, MultiIMOVO, and MultiIMOAHO, respectively, to further investigate their cooperative performance. Dietterich and Bakiri proposed the ECOC decomposition method [48] to classify multi-class datasets using error correction output coding. On the basis of ECOC, the IMECOC method developed in [56] was an improved ECOC method that simultaneously premeditated the between-class and the within-class imbalance in classifying imbalanced datasets. Furthermore, different weights were assigned to different binary classifiers in the IMECOC [56]. According to different encoding methods, IMECOC was further extended to ImECOCOVA, ImECOCsparse, ImECOCdense [34], and so on.

The above classification approaches provide a basic starting point for discovering the potential synergy between ensembles for imbalanced data and data decomposition strategies.

### 3.2. Imbalanced Learning Based on Ensemble Classification and Cost-Sensitive Scheme

By considering the cost of classifications, numerous effective cost-sensitive-based ensemble algorithms have been developed [57], which favor the minority class by assigning different weights for different samples. Ensemble classification incorporating cost-sensitive schemes can promote more robust performances than a single classification by merely combining multiple classifiers. The representative algorithm for binary classification is AdaBoost (Adaptive Boosting) [46], proposed by Freund and Schapire, in which weak classifiers were integrated to build a stronger classifier by updating weights. Given a training dataset {(*x*_1_, *y*_1_), (*x*_2_, *y*_2_), ⋯, (*x_N_*, *y_N_*)}, the weight updating rule in AdaBoost was defined as:

(5) $$D^{t+1}\left(i\right)=\frac{D^{t}\left(i\right)\exp\left(-\alpha_{t}y_{i}h_{t}\left(x_{i}\right)\right)}{Z_{t}},$$

where the initial weight takes $D^{1}\left(i\right)=1/N$, $\alpha_{t}$ is the weight parameter of the *t*-th weak classifier, $h_{t}$ is a weak classifier in the *t*-th iteration, and $Z_{t}$ is a normalization factor. The output of AdaBoost was defined as:

(6) $$H\left(x\right)=\mathrm{sign}\left(\sum_{t=1}^{T}\alpha_{t}h_{t}\left(x\right)\right).$$

To tackle multi-class classification problems, a series of AdaBoost extensions were presented. Both AdaBoost.M1 and SAMME [58] extended AdaBoost in terms of updating weights and the combination strategy of classifiers. AdaC2.M1 [59] inherited the general learning framework of AdaBoost.M1 except that it introduced misclassification costs into the weight update formula. The optimal cost setting in AdaC2.M1 was determined by employing a genetic algorithm. AdaBoost.NC [60] was an algorithm emphasizing ensemble diversity in the training process, in which a weight update rule with a penalty term was introduced. The PIBoost classifier [61] based on the ensemble method and cost-sensitivity scheme dealt with multi-class imbalanced data via a series of binary weak-learners and a margin-based exponential loss function. In addition, cost-sensitive schemes and data balancing algorithms have synergistic effects for handling imbalanced datasets in ensemble learning.

### 3.3. Imbalanced Learning Based on Ensemble Classification and Sampling Methods

For relieving the impact of the imbalanced training data on the classification model, a large number of ensemble algorithms have been improved by incorporating data-balancing algorithms. The training set is re-sampled before constructing the classification model so that the imbalance rate among various categories is close to an equilibrium. In general, ensemble methods with balancing algorithms mainly combine classifiers with under-sampling, over-sampling, or mixed sampling methods.

Under-sampling is a common method for minimizing the proportion of majority class samples in imbalanced data and improving predictive performance. Seiffert et al. [62] proposed the RUSBoost algorithm by combining the under-sampling and boosting method, which randomly removes the instances from majority classes until the desired proportion is achieved. Galar [63] presented a new EUSBoost algorithm based on RUSBoost and the random under-sampling algorithm. The diversity of base classifiers were promoted because more subclassifiers were embedded in the ensemble process. In [64], Luo et al. presented an innovative XGBoost classification method based on bagging to handle classification problems involving imbalanced data. The bagging procedure was designed with random under-sampling. XGBoost synthesizes new samples in a sufficiently small neighborhood of minority samples which averts increasing noisy samples near the classification boundary.

In terms of over-sampling, Ramentol et al. [65] construct a new synthetic minority over-sampling technique, based on the rough set theory and the lower approximation of a minority sample subset. Similarly, Douzas et al. [66] proposed an effective ensemble algorithm based on K-means clustering and SMOTE, stipulating the generation of new data in crucial areas of the space produced by clustering. The imbalance ratio, as well as the average distance among minority samples, was used as the assessment criteria to determine whether new instances should be generated.

When the imbalance rate closes to 1 after the sampling process, the dataset will achieve equilibrium. In addition, the classification accuracy is an alternative for measuring whether data strikes a balance between different classes. This was adopted by Lu et al. for designing HSBagging [67], in which a pre-processing step was conducted by using both random under-sampling and SMOTE at each bagging iteration. The classifier employed the predictive accuracy on out-of-bag instances as an optimal sampling rate for SMOTE and random under-sampling. Among classical imbalanced learning based on ensemble classification and sampling methods, UnderBagging and SMOTEBagging are used to achieve excellent performances [19]. However, HSBagging was demonstrated to show a better classification performance compared to single UnderBagging or SMOTEBagging in [67].

Some other related works based on ensemble classification and sampling methods also contribute to resolving imbalanced classification. For example, Ahmed [68] applied hybrid sampling in the RSYNBagging classifier, which considered the diversification of imbalanced data. Additionally, the ADASYNBagging algorithm [69] was coined by incorporating an algorithm and over-sampling. Although most of the above work was aimed at binary class datasets, it provided a solid foundation for the classification of multi-class imbalanced datasets. Wei et al. [70] put forward a SMOTE-decision-tree classifier that modified a binary classification algorithm for handling multi-class imbalance problems effectively.

### 3.4. Imbalanced Learning Based on Ensemble Classification and Dynamic Selection

**Figure 2:**
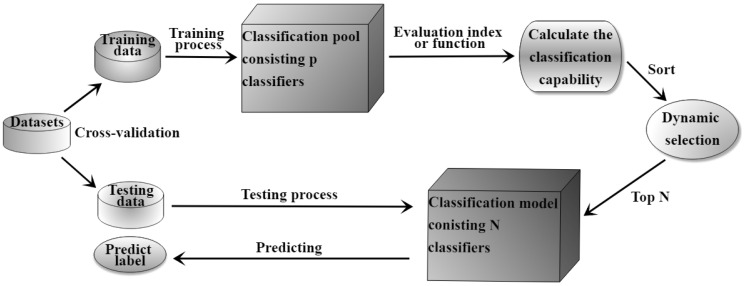
The process of dynamic ensemble classification.

With the extensive application of ensemble approaches, it has become an important issue for designing a more efficient ensemble classification algorithm. Compared with static ensemble algorithms, dynamic selection ensemble algorithms [71,72,73,74,75,76,77,78,79,80,81,82,83] have been shown to effectively improve the F-measure and G-mean values. A dynamic selection ensemble algorithm predicts the label of the test sample by evaluating the capability level of each classifier and selects the set of the most capable or competitive classifiers. In the process of dynamic ensemble classification (Figure 2), each test sample or each subset can select the optimal classification model. Generally, the selection of classifiers is realized by estimating the classification ability in the local region of the test samples or calculating the prediction accuracy of the classifiers.

A function to evaluate the classification capability can be considered as a tool to assist in the selection of classifiers. For example, Garcia et al. [84] constructed a capability function by calculating the classification score of each base classifier and selected out the top nine classifiers with the highest capability values. Specifically, the selection structure for base classifiers was a key component in their dynamic selection model. The classification accuracy can be regarded as another measure for the selection of classifiers. The approach proposed by Lin et al. [85] used a selective ensemble model to deal with multi-class classification by choosing the classifier with the highest classification accuracy in the local region of the test sample. Mendialdua et al. [86] established a more intensive approach to select classifiers for each pair of classes. The model attempted to extract the best classifier in every sub-dataset of OVO. They demonstrated that OVO and dynamic selection have a positive synergy during classification, which enabled the extension of decomposition algorithms to dynamic ensemble selection strategies. Woloszynski et al. [87] used a probability model to evaluate the classification ability of the base classifiers and introduced a random reference classifier in the process of ensemble classification. The probability of the correct classification of the random reference classifier was employed as the measure of the competence of the actual classifier, which combined the dynamic selection of the classifiers with the probabilistic method.

In addition to the selection manner of classifiers, base classifiers’ generalization ability also has an extremely important impact on dynamic selection results. Cruz et al. [88] developed an improved dynamic selection approach. In the first stage, prototype selection techniques were applied to the training data to reduce the overlap between classes. In the next generalization process, a local adaptive K-nearest neighbor algorithm was adopted to minimize the influence of noisy samples on the competency area. Meanwhile, they demonstrated that the distribution of imbalanced datasets would directly affect selecting the optimal classifier during the dynamic selection process, and datasets with a complex structure would result in poor classification. Focusing on the complex structure of imbalanced data, Brun et al. [89] selected the classifier trained on the data subset whose complexity is similar to the neighborhood of the test instances. They also conducted an in-depth consideration and analysis of the data structure in the field of dynamic ensemble learning. Cruz et al. developed a novel dynamic selection framework [90] which extracted meta-features from the training data for training meta-classifiers to judge whether the base classifier had the sufficient ability to classify test instances.

Although each of the strategies mentioned above has its own merits and improves the performance in the design of classifiers, there is still room for improvement in terms of performance optimization by designing dynamic selection in multi-class imbalanced classification. Inspired by the related literature, this study focuses on the investigation of the classification performance of classic multi-classification algorithms combining dynamic selection approaches.

## 4. Experimental Comparison of Multi-Class Imbalanced Classifiers by Incorporating Dynamic Selection

Due to the significant advantages of ensemble classification algorithms in dealing with class imbalance, this study merges dynamic selection with popular ensemble classification algorithms for multi-class datasets, aimed towards verifying the effectiveness of dynamic selection.

### 4.1. Experimental Procedure

We employ a homogeneous classifier to generate the candidate classifier pool, and 14 multi-class imbalance ensemble classification algorithms (Figure 1) are employed as base classifiers, respectively. By combining the above-mentioned base classifiers and the dynamic selection process proposed in [84], dynamic ensemble classifiers are designed for both binary class and multi-class imbalanced datasets. The overall process of dynamic selection is shown in Figure 2. The function for capability evaluation referred to in [84] is defined as follows.

Given a test sample $x_{i}$ and a classifier *h*, we calculate the classification capability of classifier *h* for $x_{i}$:

(7) $$\begin{array}{ccc} & & F_{h|x_{i}}=\sum_{t=1}^{k}I\left(x_{it}\right)\times w_{it},\\ & & I\left(h\left(x_{it}\right)=y_{t}\right)=\left\{\begin{array}{cc} 0, & h\left(x_{it}\right)\neq y_{t},\\ 1, & h\left(x_{it}\right)=y_{t}, \end{array}\right. \end{array}$$

where *k* is the number of nearest neighbor instances of $x_{i}$ in the training data; $x_{it}$ is the *t*-th neighbor instance of $x_{i}$; $y_{t}$ is the true label of instance $x_{it}$; $w_{it}$ is the weight of nearest neighbor sample $x_{it}$, $w_{it}=\frac{1}{1+exp(\lambda *m)}$, λ is the scaling coefficient, and *m* is the number of samples with the same class as $x_{it}$. Obviously, the more samples with the same class as $x_{it}$, the lower the weight, which indirectly increases the weight of a minority sample. Meanwhile, if the predicted labels of the K-nearest neighbor samples are identical to the true label, *I* returns 1; otherwise, *I* returns 0. The classification capability of classifier *h* for $x_{i}$ is reflected by the classification performance of its neighbor samples. Taking AdaBoostNC as the base classifier, the procedure of the dynamic AdaBoostNC model is described in Algorithm 1. The other 13 dynamic ensemble algorithms are constructed similarly.
**Algorithm 1:** Dynamic AdaBoostNC classifier.
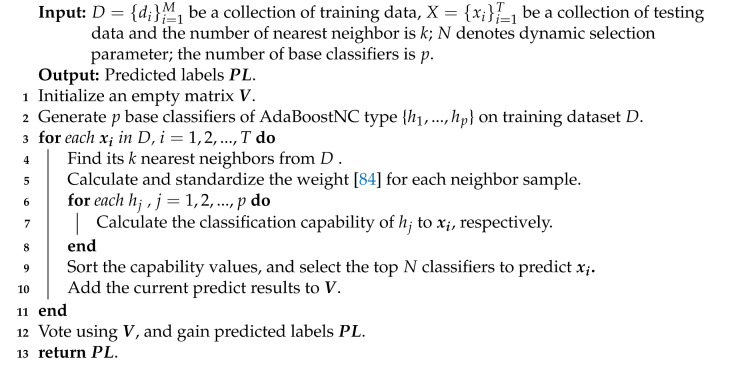


The datasets for the experiments come from the four repositories mentioned in Table 1, including 56 datasets, 32 of which are suitable for binary classification, and the other 24 datasets are available for multi-class classification. These data are closely related to the fields of life sciences, medical research, bank credit evaluation, network fault detection, etc. More information concerning these datasets is offered in Table 2 and Table 3. As can be seen from these tables, the experimental datasets are diverse in the number of attributes (4∼40), classes (2∼10), continuous or discrete attributes, class distribution, and imbalance ratio (1.003∼853).

In this study, 14 groups of comparative experiments between dynamic ensemble algorithms and state-of-the-art methods are carried out on both binary and multi-class imbalanced datasets. In the experimentation, the nearest neighbor number *k* is set to 9. The dynamic selection parameter *N* takes 9 (the settings for both parameters are based on reference [84]), the number of base classifiers *p* is chosen to be 16. Five-fold cross-validation is carried out for all methods for each dataset. The average values of 5 runs of experimental results are calculated for obtaining the predictive performance of the designed methods.

### 4.2. Experiments Results for Binary Class Datasets

**Figure 3:**
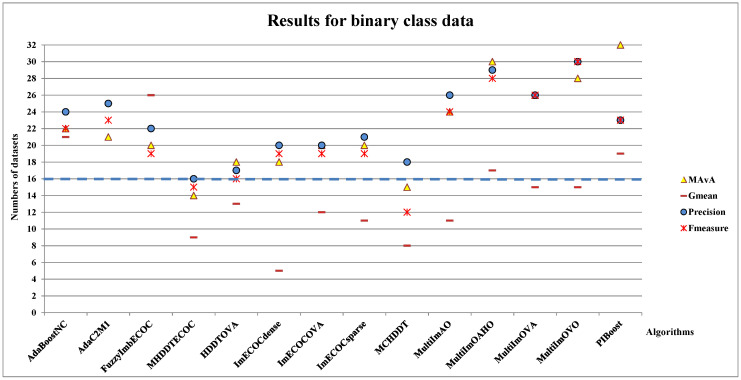
The total number of binary class imbalanced datasets on which prediction results are improved after adding the dynamic selection.

This study first examines the performance of the designed dynamic model on 32 binary class datasets. Aiming to illustrate the performance of the classifier more distinctly, we compare the MAvA, G-mean, precision, and F-measure results with classic algorithms before and after adding the dynamic selection process (Figure 3), where the dashed line at “Number of datasets = 16” means half of the number of binary datasets, and the points represent numbers of datasets on which prediction results are improved after dynamic selection. If the points are above the dashed line, it indicates that the dynamic selection effect on the corresponding index is satisfactory, i.e., the performance of the dynamic selection ensemble classifiers are better than original algorithms and vice versa.

We observe from Figure 3 that by incorporating with the dynamic selection strategy, the results of 12 classical imbalanced classification algorithms are improved under the MAvA, precision, and F-measure indicators, which mean higher precision is obtained for more than half of the binary datasets. The effect of dynamic selection is not obvious for MHDDTECOC and HDDTOVA. The points are below the dashed line under MAvA and F-measure indicators, which indicate the higher results only shown on individual datasets. However, other results for 12 classical imbalanced classification algorithms are over the dashed line except the above two classification algorithms, which indicate incorporating dynamic selection can promote predictive performance (MAvA, precision, and F-measure) for binary class datasets.

### 4.3. Experiments Results for Multi-Class Imbalanced Datasets

**Figure 4:**
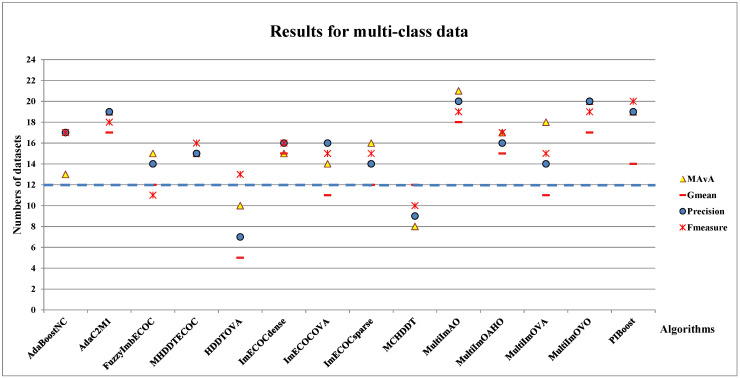
The total number of multi-class imbalanced datasets on which prediction results are improved after adding the dynamic selection.

**Figure 5:**
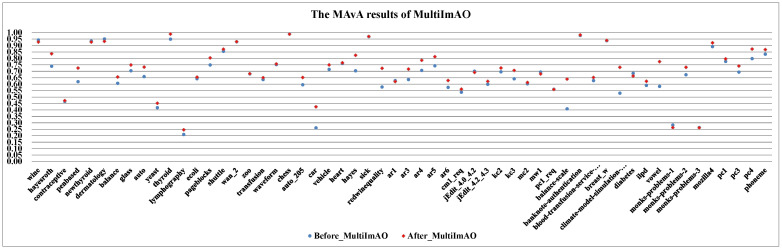
The MAvA results of MultiImAO for 56 datasets.

**Figure 6:**
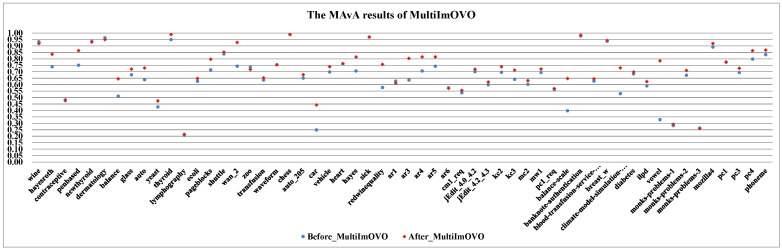
The MAvA results of MultiImOVO for 56 datasets.

**Figure 7:**
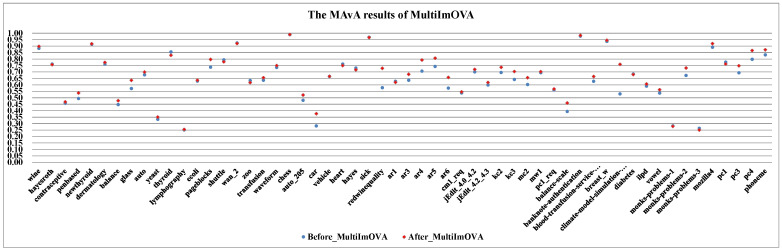
The MAvA results of MultiImOVA for 56 datasets.

**Figure 8:**
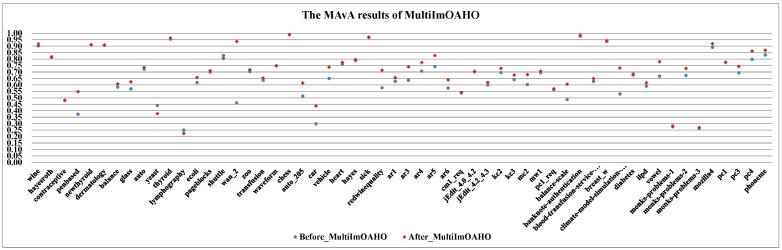
The MAvA results of MultiImOAHO for 56 datasets.

In this study, the above-mentioned classifiers are tested on 24 multi-class imbalanced datasets, where the dashed line at “Number of datasets = 12” means half of the number of multi-class datasets. The multi-class results are shown in Figure 4, and the main observations are enumerated as follows:

(I) The effect of dynamic selection for MCHDDT and HDDTOVA are not satisfactory (the points of 3 indicators are below the dashed line), and the performance of the other 12 dynamic selection ensemble classifiers are better than the original algorithms. The MAvA, precision, and F-measure indicators of the improved classifiers have been distinctly improved after dynamic selection. However, due to the extremely low representation of the minority samples, the recognition rate for the minority category may drop sharply, resulting in a lower G-mean value. In fact, 11 of the classifiers demonstrate favorable G-mean results on more than half of the multi-class datasets after syncretizing with dynamic selection. In this regard, it is obvious that dynamic selection models for multi-class imbalanced datasets showed superior characteristics compared to those for binary data.
(II) By combining dynamic selection algorithms, MultiImOVA, MultiImOVO, MultiImOAHO, and MultiImAO have exceptional performance, on which predictive performances are effectively improved compared to using a single classification algorithm, on the whole. Therefore, we further validate the conclusion that data decomposition techniques and dynamic selection have a positive synergy during classification [86]. In particular, the classification MAvA values of the above four algorithms on all 56 datasets are shown in Figure 5, Figure 6, Figure 7 and Figure 8.
  The results indicate that dynamic selection provides potential strategies for dealing with imbalanced datasets covering binary class and multi-class imbalanced datasets. Moreover, for the same dataset, we have counted the number of classification algorithms with improved classification results after dynamic selection. As shown in Table 4, Table 5, Table 6 and Table 7, regardless of the structure of the data (both binary class and multi-class imbalanced data), the classification algorithms, for the most part, can better classify imbalanced data after employing dynamic selection. The results reveal that incorporating dynamic selection can relieve the impact of imbalanced training data on the classifier performance. Therefore, a dynamic selection ensemble algorithm can be a potential solution for the imbalanced classification problem.

## 5. Patch-Ensemble Classification for Imbalanced Data

**Figure 9:**
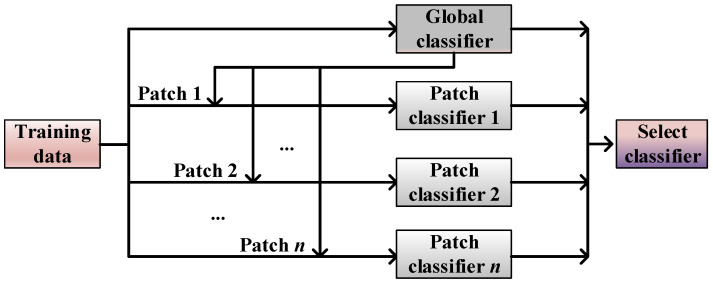
Patch classifier.

Dynamic selection strategies and ensemble classification algorithms have a synergistic effect in classification. Training a dynamic selection ensemble classification scheme with excellent performance is usually an uncertain task, which depends on previous experience and a trial-and-error experiment process. Patch Learning (PL), proposed by Wu et al. [91], is a new machine learning strategy for solving the fitting problem in classification. As shown in Figure 9, patch learning is a combination of parallel and serial models, which focuses on the misclassified samples during the training procedure, and enhances the classification diversity with the construction of multiple local classifiers [91]. In this study, a patch-ensemble classification method is designed for classifying imbalanced data, which connects patch learning with a dynamic selection ensemble classification scheme.

### 5.1. Patch Learning

Patch learning consists mainly of the following three steps [91]:

(I) Train an initial global model using all the training data;
(II) Select the incorrectly classified training data to construct several local patch models;
(III) The correctly classified training samples are utilized to update the global model.

For a new testing sample, PL firstly determines if the sample belongs to a patch so that the corresponding local patch model is selected for a classification task. Otherwise, the global model is employed.

### 5.2. Patch-Ensemble Classifier

In classification, a classifier tries to distinguish boundaries between binary or multi-class. If the training samples belong to the same class, we can directly detect whether a new testing sample belongs to this class during the predicted phase. One-Class SVM can better solve the above problem [92]. Considering that in patch learning, when the testing sample selects the global classifier or several patch classifiers, it is necessary to detect the similarity between the samples and several patches. The ensemble classification method with patch designed in this paper uses the existing imbalanced ensemble classification algorithm as the global classifier and One-Class SVM as the patch classifier for experimental design. The specific process is shown in Algorithm 2.

In the training process, the number of patch classifiers is determined according to the number of classes misclassified by the global classifier. To ensure that the global classifier maintains its best classification effect for multi-class imbalanced datasets, we weaken the boundary between global and patch training data allowing the training samples of the global classifier to partly overlap with that of the patch classifier. During the testing process, the distances between the new sample and various center points of the training sample are calculated, used as the selection condition to dynamically choose a patch classifier or a global classifier for dealing with the testing data.

### 5.3. Experiments and Analysis

To explore the effectiveness of patch ensemble classifiers for imbalanced classification, in this study, the patch ensemble classifier is compared with the classical imbalance ensemble algorithms. We choose the AdaC2M1 algorithm as the global classifier and One-Class SVM as the patch classifier, and the relevant experimental data are detailed in Table 2 and Table 3. The performance of the designed patch ensemble classifiers are evaluated by five-fold cross validation on multiple data sets. The results of the designed patch ensemble classifier in this paper are compared with the top five of 14 classical imbalance ensemble algorithms. To show the results more clearly, the classification results of each method are sorted as a whole. The times of dominant classification results of each algorithm in 56 datasets are counted respectively under MAVA, Precision, G-Mean, and F-Measure indexes.
**Algorithm 2:** Patch ensemble classifier.
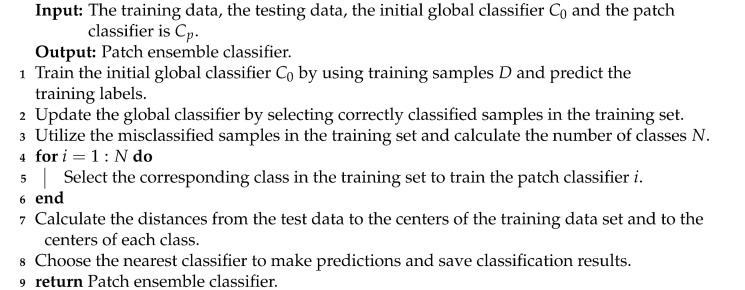


As shown in Table 8, Table 9, Table 10 and Table 11, the proposed patch ensemble classification method achieves better MAvA, Precision, and F-measure values in more than half of the datasets. If only the top two results of the classification are considered, the same conclusion can be drawn. Under the G-mean indicator, the proposed method obtains the optimal and suboptimal classification results on 12 imbalanced datasets, which has a certain degree classification potential compared with other methods.

## 6. Conclusions

In this study, we reviewed state-of-the-art ensemble classification algorithms for imbalanced data and compared the performance of 14 existing multi-class imbalanced classification algorithms by incorporating a dynamic selection strategy. By applying these dynamic imbalance ensemble classifiers to 56 public datasets, the experimental results demonstrate that the dynamic ensemble classification methods obtain significantly better MAvA, precision, G-mean, and F-measure performances than the original 14 algorithms. In particular, dynamic multi-class ensemble classifiers have the potential to achieve an ideal identification performance. We also designed a patch ensemble classification method, which uses misclassified samples to train patch classifiers for increasing classification diversity. Experiments showed that this method has a certain classification potential for multi-class imbalanced classification. In future work, we will consider the imbalance ratio to further improve the classification performance and tackle practical problems under its guidance.

## Figures and Tables

**Table 1:** Sources of imbalanced datasets.

Name	Source
UCI	https://archive.ics.uci.edu/ml/index.php (accessed on 9 November 2019)
OpenML	https://www.openml.org (accessed on 20 March 2020)
KEEL	https://sci2s.ugr.es/keel/imbalanced.php (accessed on 7 January 2020)
DefectPrediction	http://tunedit.org/repo/PROMISE/DefectPrediction (accessed on 10 April 2020)

**Table 2:** Description of binary datasets.

Datasets	Number of Data	Number of Attribute	Continuous Attribute	Discrete Attribute	Classes Distribution	Classes	Imbalance Ratio
transfusion	748	4	4	0	178, 570	2	3.202
heart	270	13	13	0	150, 120	2	1.250
chess	3196	35	35	0	1669, 1527	2	1.093
sick	2800	27	27	0	2629, 171	2	15.374
redwinequality	691	11	11	0	681, 10	2	68.100
ar1	121	29	9	20	112, 9	2	12.444
ar3	63	29	9	20	55, 8	2	6.875
ar4	107	29	9	20	87, 20	2	4.350
ar5	36	29	9	20	28, 8	2	3.500
ar6	101	29	9	20	86, 15	2	5.733
cm1_req	89	8	0	8	69, 20	2	3.450
jEdit_4.0_4.2	274	8	0	8	140, 134	2	1.045
jEdit_4.2_4.3	369	8	0	8	165, 204	2	1.236
kc2	522	21	7	14	415, 107	2	3.879
kc3	458	39	14	25	415, 43	2	9.651
mc2	161	39	15	24	109, 52	2	2.096
mw1	403	37	13	24	372, 31	2	12.000
pc1_req	320	8	0	8	213, 107	2	1.991
banknote-authentication	1372	4	4	0	762, 610	2	1.249
blood-transfusion-service-center	748	4	0	4	570, 178	2	3.202
breast_w	699	9	0	9	458, 241	2	1.900
climate-model-simulation-crashes	540	20	17	3	46, 494	2	10.739
diabetes	768	8	2	6	268, 500	2	1.866
ilpd	583	9	5	4	416, 167	2	2.491
monks-problems1	556	6	0	6	272, 284	2	1.044
monks-problems2	601	6	0	6	300, 301	2	1.003
monks-problems3	554	6	0	6	275, 279	2	1.015
mozilla4	15,545	4	0	4	10,437, 5108	2	2.043
pc1	1109	21	17	4	77, 1032	2	13.403
pc3	1563	37	14	23	160, 1403	2	8.769
pc4	1458	37	12	25	178, 1280	2	7.191
phoneme	5404	5	5	0	3818, 1586	2	2.407

**Table 3:** Description of multi-class imbalanced datasets.

Datasets	Number of Data	Number of Attribute	Continuous Attribute	Discrete Attribute	Classes Distribution	Classes	Imbalance Ratio
wine	178	13	11	2	59, 71, 48	3	1.479
hayesroth	132	4	0	4	51, 51, 30	3	1.700
contraceptive	1473	9	0	9	629, 333, 511	3	1.889
penbased	1100	16	0	16	114, 114, 106, 114, 106, 105, 115, 105, 106, 115	10	1.095
newthyroid	215	5	4	1	150, 35, 30	3	5.000
dermatology	366	34	0	34	112, 61, 72, 49, 52, 20	6	5.600
balance	625	4	0	4	49, 288, 288	3	5.878
glass	214	9	9	0	70, 76, 17, 13, 9, 29	6	8.444
auto	406	7	1	6	254, 73, 79	3	3.479
yeast	1484	9	9	0	288, 480, 626, 35, 30, 20, 5	7	125.200
thyroid	720	21	6	15	17, 37, 666	3	39.176
lymphography	148	18	0	18	57, 37, 18, 10, 8, 8, 8, 2	8	28.500
ecoli	336	7	7	0	143, 77, 2, 2, 35, 20, 5, 52	8	71.500
pageblocks	548	10	4	6	492, 33, 3, 8, 12	5	164.000
shuttle	2175	9	0	9	1706, 2, 6, 338, 123	5	853.000
wan_2	750	2	2	0	100, 150, 250, 250	4	2.500
zoo	101	16	0	16	41, 20, 5, 13, 4, 8, 10	7	10.250
hayes	132	5	0	5	51, 51, 30	3	1.700
waveform	5000	40	40	0	169, 1653, 1655	3	1.024
auto_205	205	25	18	7	54, 32, 27, 67, 22, 3	6	22.333
car	1728	6	6	0	1210, 384, 69, 65	4	18.615
vehicle	846	18	18	0	217, 217, 216, 196	4	1.107
balance-scale	625	4	0	4	49, 288, 288	3	5.878
vowel	990	10	10	0	180, 180, 180, 90, 180, 180	6	2.000

**Table 4:** Numbers of improved classification algorithms after dynamic selection under the MAvA index.

Datasets	Number	Datasets	Number	Datasets	Number
wine	4	ar3	8	shuttle	6
hayesroth	7	ar4	10	wan_2	7
contraceptive	10	ar5	9	zoo	14
penbased	12	ar6	12	transfusion	14
newthyroid	10	cm1_req	10	waveform	14
dermatology	10	jEdit_4.0_4.2	6	chess	13
balance	6	jEdit_4.0_4.3	11	auto_205	13
glass	12	kc2	7	car	11
auto	9	kc3	9	vehicle	9
yeast	8	mc2	13	heart	13
thyroid	12	mw1	9	hayes	11
lymphography	10	pc1_req	5	sick	13
ecoli	11	balance-scale	7	redwinequality	8
pageblocks	7	banknote-authentication	2	ar1	12
blood-transfusion-service-center	9	breast_w	9	climate-model-simulation-crashes	14
diabetes	7	ilpd	10	vowel	14
monks-problems1	2	monks-problems2	10	monks-problems3	2
mozilla4	10	pc1	9	pc3	6
pc4	10	phoneme	12		

**Table 5:** Numbers of improved classification algorithms after dynamic selection under the G-mean index.

Datasets	Number	Datasets	Number	Datasets	Number
wine	8	ar3	11	shuttle	10
hayesroth	9	ar4	11	wan_2	7
contraceptive	10	ar5	5	zoo	14
penbased	12	ar6	13	transfusion	11
newthyroid	11	cm1_req	12	waveform	13
dermatology	11	jEdit_4.0_4.2	6	chess	12
balance	8	jEdit_4.0_4.3	13	auto_205	13
glass	9	kc2	10	car	10
auto	9	kc3	10	vehicle	9
yeast	9	mc2	14	heart	8
thyroid	13	mw1	11	hayes	12
lymphography	0	pc1_req	8	sick	11
ecoli	8	balance-scale	8	redwinequality	12
pageblocks	12	banknote-authentication	5	ar1	12
blood-transfusion-service-center	9	breast_w	9	climate-model-simulation-crashes	13
diabetes	7	ilpd	10	vowel	14
monks-problems1	2	monks-problems2	10	monks-problems3	2
mozilla4	10	pc1	9	pc3	7
pc4	10	phoneme	12		

**Table 6:** Numbers of improved classification algorithms after dynamic selection under the Precision index.

Datasets	Number	Datasets	Number	Datasets	Number
wine	4	ar3	7	shuttle	7
hayesroth	7	ar4	11	wan_2	9
contraceptive	10	ar5	6	zoo	14
penbased	13	ar6	13	transfusion	11
newthyroid	9	cm1_req	10	waveform	10
dermatology	5	jEdit_4.0_4.2	5	chess	11
balance	4	jEdit_4.0_4.3	11	auto_205	12
glass	11	kc2	8	car	13
auto	9	kc3	8	vehicle	9
yeast	10	mc2	12	heart	13
thyroid	10	mw1	9	hayes	13
lymphography	11	pc1_req	7	sick	11
ecoli	11	balance-scale	6	redwinequality	9
pageblocks	5	banknote-authentication	7	ar1	11
blood-transfusion-service-center	9	breast_w	9	climate-model-simulation-crashes	13
diabetes	6	ilpd	10	vowel	14
monks-problems1	2	monks-problems2	10	monks-problems3	2
mozilla4	10	pc1	8	pc3	5
pc4	9	phoneme	12		

**Table 7:** Numbers of improved classification algorithms after dynamic selection under the F-measure index.

Datasets	Number	Datasets	Number	Datasets	Number
wine	4	ar3	7	shuttle	7
hayesroth	7	ar4	9	wan_2	7
contraceptive	9	ar5	8	zoo	9
penbased	13	ar6	11	transfusion	12
newthyroid	10	cm1_req	10	waveform	5
dermatology	5	jEdit_4.0_4.2	4	chess	12
balance	10	jEdit_4.0_4.3	12	auto_205	11
glass	11	kc2	9	car	12
auto	9	kc3	8	vehicle	9
yeast	10	mc2	11	heart	12
thyroid	11	mw1	8	hayes	8
lymphography	7	pc1_req	5	sick	10
ecoli	13	balance-scale	11	redwinequality	11
pageblocks	7	banknote-authentication	11	ar1	11
blood-transfusion-service-center	9	breast_w	9	climate-model-simulation-crashes	12
diabetes	7	ilpd	10	vowel	14
monks-problems1	2	monks-problems2	10	monks-problems3	2
mozilla4	10	pc1	9	pc3	6
pc4	10	phoneme	12		

**Table 8:** MAvA results.

	Patch Ensemble	A	B	C	D	E	F	G	H	I	G	K	L	M	N
Times of the optimal MAvA	7	1	3	8	1	1	3	2	4	3	7	8	8	7	5
Times of the second MAvA	7	3	8	3	8	1	4	3	6	2	5	4	3	5	1
Times of the third MAvA	7	1	12	1	4	4	7	4	6	4	2	2	3	2	1
Total	**21**	5	**23**	12	13	6	14	9	16	9	14	14	14	14	7

1. The 14 classical imbalanced classification algorithms are AdaBoostNC (A), AdaC2M1 (B), FuzzyImbECOC (C), MHDDTECOC (D), HDDTOVA (E), ImECOCdense (F), ImECOCOVA (G), ImECOCsparse (H), MCHDDT (I), MultiIMAO (J), MultiIMOAHO (K), MultiIMOVA (L), MultiIMOVO (M), and Piboost (N). 2. “Times of the optimal MAvA” in the table represents the times of best classification result of each algorithm in 56 datasets. “Times of the second MAvA” in the table represents the times of suboptimum classification result of each algorithm, and so on. 3. The bold number indicates that the classification result is equal to or better than that of the Patch Ensemble classifier.

**Table 9:** G-mean results.

	Patch Ensemble	A	B	C	D	E	F	G	H	I	G	K	L	M	N
Times of the optimal MAvA	6	3	5	8	0	0	2	2	3	1	10	10	9	9	3
Times of the second MAvA	6	3	5	3	7	1	6	5	8	3	7	5	5	7	0
Times of the third MAvA	6	1	9	1	6	4	11	7	8	5	4	5	4	4	2
Total	**18**	7	**19**	12	13	5	**19**	14	**19**	9	**21**	**20**	**18**	**20**	5

The bold number indicates that the classification result is equal to or better than that of the Patch Ensemble classifier.

**Table 10:** Precision results.

	Patch Ensemble	A	B	C	D	E	F	G	H	I	G	K	L	M	N
Times of the optimal MAvA	4	2	4	3	4	2	0	0	0	3	0	1	0	0	8
Times of the second MAvA	10	2	6	3	2	0	3	0	1	2	0	0	0	0	1
Times of the third MAvA	8	4	8	0	4	4	2	1	5	3	0	0	2	0	1
Total	**22**	8	18	6	10	6	5	1	6	8	0	1	2	0	10

The bold number indicates that the classification result is equal to or better than that of the Patch Ensemble classifier.

**Table 11:** F-measure results.

	Patch Ensemble	A	B	C	D	E	F	G	H	I	G	K	L	M	N
Times of the optimal MAvA	3	3	2	4	3	5	0	0	0	5	0	0	0	0	12
Times of the second MAvA	7	2	7	2	8	2	3	1	2	3	0	0	0	0	1
Times of the third MAvA	9	2	10	1	7	4	0	0	2	6	1	0	0	1	0
Total	**19**	7	**19**	7	18	11	3	1	4	14	1	0	0	1	13

The bold number indicates that the classification result is equal to or better than that of the Patch Ensemble classifier.

## Data Availability

The raw experimental is available at the following website: https://archive.ics.uci.edu/ml/index.php, https://www.openml.org, https://sci2s.ugr.es/keel/imbalanced.php, and http://tunedit.org/repo/PROMISE/DefectPrediction (all accessed on 24 June 2021).
